# Mutations in *SLC25A22*: hyperprolinaemia, vacuolated fibroblasts and presentation with developmental delay

**DOI:** 10.1007/s10545-017-0025-7

**Published:** 2017-03-02

**Authors:** Emma S. Reid, Hywel Williams, Glenn Anderson, Malika Benatti, Kling Chong, Chela James, Louise Ocaka, GOSgene, Cheryl Hemingway, Daniel Little, Richard Brown, Alasdair Parker, Simon Holden, Emma Footitt, Shamima Rahman, Paul Gissen, Philippa B. Mills, Peter T. Clayton

**Affiliations:** 1 Centre for Translational Omics, Genetics and Genomic Medicine, UCL Great Ormond Street Institute of Child Health, 30 Guilford Street, London, WC1N 1EH UK; 2 Histopathology Department, Great Ormond Street Hospital NHS Foundation Trust, Great Ormond Street, London, WC1N 3JH UK; 3 Radiology Department, Great Ormond Street Hospital NHS Foundation Trust, Great Ormond Street, London, WC1N 3JH UK; 4 Neurology Department, Great Ormond Street Hospital NHS Foundation Trust, Great Ormond Street, London, WC1N 3JH UK; 5 The Medical Research Council Laboratory for Molecular Cell Biology, University College London, Gower Street, London, WC1E 6BT UK; 6 Peterborough and Stamford Hospitals NHS Foundation Trust, Edith Cavell Campus, Bretton Gate, Peterborough, PE3 9GZ UK; 7 Child Development Centre, Addenbrooke’s Hospital, Cambridge, CB2 0QQ UK; 8 Clinical Genetics, Addenbrooke’s Hospital, Cambridge, CB2 0QQ UK; 9 Metabolic Medicine Department, Great Ormond Street Hospital NHS Foundation Trust, Great Ormond Street, London, WC1N 3JH UK; 10 Mitochondrial Research Group, Genetics and Genomic Medicine, UCL Great Ormond Street Institute of Child Health, 30 Guilford Street, London, WC1N 1EH UK

## Abstract

Mutations in *SLC25A22* are known to cause neonatal epileptic encephalopathy and migrating partial seizures in infancy. Using whole exome sequencing we identified four novel *SLC25A22* mutations in six children from three families. Five patients presented clinical features similar to those in the literature including hypotonia, refractory neonatal-onset seizures and developmental delay. However, the sixth patients presented atypically with isolated developmental delay, developing late-onset (absence) seizures only at 7 years of age. Abnormal metabolite levels have not been documented in the nine patients described previously. One patient in our series was referred to the metabolic clinic because of persistent hyperprolinaemia and another three had raised plasma proline when tested. Analysis of the post-prandial plasma amino acid response in one patient showed abnormally high concentrations of several amino acids. This suggested that, in the fed state, when amino acids are the preferred fuel for the liver, trans-deamination of amino acids requires transportation of glutamate into liver mitochondria by SLC25A22 for deamination by glutamate dehydrogenase; SLC25A22 is an important mitochondrial glutamate transporter in liver as well as in brain. Electron microscopy of patient fibroblasts demonstrated widespread vacuolation containing neutral and phospho-lipids as demonstrated by Oil Red O and Sudan Black tinctorial staining; this might be explained by impaired activity of the proline/pyrroline-5-carboxylate (P5C) shuttle if SLC25A22 transports pyrroline-5-carboxylate/glutamate-γ-semialdehyde as well as glutamate.

**Electronic supplementary material:**

The online version of this article (doi:10.1007/s10545-017-0025-7) contains supplementary material, which is available to authorized users.

## Introduction

SLC25A22 deficiency causes neonatal epileptic encephalopathy and migrating partial seizures in infancy. In nine patients (Molinari et al 2005; Molinari et al 2009; Poduri et al 2013; Cohen et al 2014), no abnormalities of routine metabolic investigations were reported. Patients presented with refractory seizures, hypotonia, visual inattention with evidence of post-retinal dysfunction, microcephaly, a burst-suppression pattern on EEG and hypoplasia of the cerebellum and corpus callosum on MRI.

SLC25A22 is a mitochondrial glutamate transporter (Fiermonte et al 2002). In liposomes it catalyses glutamate exchange (bidirectional transport) and uniport (Fiermonte et al 2002). Digitonin-permeabilised fibroblasts from patients with *SLC25A22* mutations show reduced oxidation of glutamate (Molinari et al 2005). Given the widespread expression of *SLC25A22*, it is surprising that no amino acid abnormalities were reported. We present six patients with novel homozygous/compound heterozygous mutations in *SLC25A22*. The first was referred because of hyperprolinaemia; for three others, first-line biochemical screening showed hyperprolinaemia. All three patients tested showed lipid vacuoles in fibroblasts.

## Materials and methods

This study was approved by the National Research Ethics Service Committee, Bloomsbury (13/LO/0168).

### Chemical reagents

Cell culture reagents were from Life Technologies (Paisley, UK); other chemicals were from Sigma Aldrich (Gillingham, UK).

### Genetic analysis

Whole exome sequencing (WES) of DNA from blood was performed for patients 1 and 4–6 (BGI Genomics, Hong Kong). DNA, captured using the Agilent SureSelect v4 (51 Mb) kit (Santa Clara, CA), was sequenced on an Illumina HiSeq 2000 platform (San Diego, CA). Data was aligned to the human_g1k_v37 genome and analysed using Ingenuity Variant Analysis software (Redwood City, CA) (Reid et al 2015). Prioritisation of variants was based on: candidate genes for phenotype, predicted effect on gene function, conservation of amino acid position and frequency of the variant in the databases including the 1000 genomes, Ensembl and the Human Gene Mutation Database*. SIFT* and PolyPhen-2* were used to predict pathogenicity. Sanger sequencing was used to confirm *SLC25A22* mutations and for prenatal diagnoses (PND). SLC25A22 sequences from different species (accession numbers in Supplementary Methods) were aligned using ClustalW2* (Thompson et al 1994). The human reference sequence used was NM_024698.5)

*URLs follow References

### Fibroblast culture and tinctorial staining

Fibroblasts were grown in Ham’s F10 medium containing 10% fetal bovine serum, penicillin (100 U/L) and streptomycin (100 μg/mL) at 37 °C in 5% carbon dioxide. Once 80% confluent, fibroblasts were detached using 0.25% trypsin-EDTA, resuspended in Ham’s F10 medium and approximately 8x10^6^ cells were immobilised as a monolayer on glass slides using a Cytospin 4 (Thermo Scientific). Cells were allowed to air-dry, stained using H&E, Sudan Black, Oil Red O and Luxol Fast Blue (Suvarna et al 2012) and visualised using a Nikon Optiphot microscope; images were captured using a Leica DMD108.

### Nucleoporin (p62) immunofluorescence and quantification

Nucleoporin (p62) immunofluorescence was performed as in Yasin et al (2013) and CellProfiler 2.1.1 (Carpenter et al 2006) was used to analyse the images obtained (Supplementary Methods).

### Electron microscopy (EM)

EM was undertaken essentially as in Yasin et al (2013) (Supplementary Methods).

## Results

### Case studies

#### Patients 1–3

A 2y old boy (patient 1) presented to the metabolic clinic with epilepsy, severe developmental delay and hyperprolinaemia. His parents are first cousins of Afghan origin. From birth he had central and peripheral hypotonia and delayed feeding. At 6 wks he developed myoclonic and tonic-clonic seizures, dystonic movements and was not fixing and following. At 2y he showed severe global developmental delay, visually unresponsive behaviour with impaired cortical responses to light and myoclonic and generalised tonic-clonic seizures. MRI showed frontotemporal hypoplasia, globally delayed myelination, delayed temporal pole myelination, prominent cerebellar folia and a small splenium. Extensive investigations were undertaken (Supplementary Tables 1–4, 6). Amino acid profiles were consistently abnormal in plasma, urine and CSF. Plasma proline was 322–1195 μmol/L (*n* = 11; ref: 85–290 μmol/L). At 2 y 8 mo, plasma amino acids were assayed after a 6 hr fast and 1 hr, 2 hr and 4 hr post-prandially (Supplementary Table 2). A muscle biopsy showed increased lipid and glycogen but no ragged-red or cytochrome oxidase-negative fibres, and normal activities of respiratory chain complexes. Ammonia was slightly raised once (50; ref: <40 μmol/L) and white cell ubiquinone was low (24; ref: 37–133 pmol/mg). At 7 y, seizures were well controlled on levetiracetam (40 mg/kg/day), sodium valproate (20 mg/kg/day) and pyridoxine (15 mg bd). At 7 y 11 mo, he was wheelchair-bound and severely developmentally delayed, although continuing to make slow progress. Eye movements were full but he was not fixing or following. He had no dystonic movements, and reflexes were elicitable in his upper limbs only.

In the mother’s next pregnancy, PND revealed the homozygous *SLC25A22* mutation and the parents elected to terminate. The following pregnancy was a twin pregnancy and PND indicated that both fetuses (patients 2 and 3) had homozygous *SLC25A22* mutations; this time the parents opted not to terminate. As their affected brother had a low white cell ubiquinone, the twins were commenced on ubiquinone (10 mg/kg/day) from birth. Initially they appeared less severely affected than their brother. Whereas at 6 wks patient 1 was profoundly hypotonic, visually inattentive and had seizures and dystonic movements, at 6 wks the twins had milder, and mainly axial, hypotonia. They were both smiling and fixing and had no abnormal movements. Both had a smooth philtrum and low set ears. At 16 wks, both twins developed seizures with a flexor spasm-like semiology and multifocal EEG abnormalities; levetiracetam (40 mg/kg/day) was started. At 12 mo, both twins have generalised tonic-clonic seizures twice a week and have developmental stasis. Patient 2 occasionally fixes and smiles and patient 3 occasionally fixes but does not smile. They are hypotonic with no head control but normal reflexes. Plasma amino acids were measured on two occasions and at 11 mo patient 2 showed a proline of 418 μmol/L (ref: 85–290 μmol/L), alongside other mild abnormalities (Supplementary Table 2). Other plasma proline results for patients 2 and 3 were within the normal range.

#### Patients 4 and 5

Patient 4, a 6 y old girl, presented with epilepsy and severe developmental delay. Her parents are first cousins of Syrian origin. She has a healthy older brother and an affected younger sister. Within the first month of life she developed a febrile illness followed by seizures, treated with sodium valproate with some clinical improvement. Her current treatment is sodium valproate 30 mg/kg/day and clonazepam 500 μg nocte. She continues to have brief (<1 min) seizures at least weekly—usually tonic seizures and sometimes in clusters. She started crawling and sitting independently at 2 y 6 mo. At 7 y she could babble but had no words. Developmental progress was very slow but she had not regressed. She was globally hypotonic although lower limb reflexes were brisk. At 6 y, an EEG showed symmetrical diffuse irregular slow wave activity and MRI showed symmetrical signal abnormalities of the insular cortex and adjacent capsular white matter, delayed temporal pole myelination, prominent cerebellar folia and a small splenium. Plasma proline was consistently > 350 μmol/L (*n* = 2; ref: 85–290 μmol/L).

Patient 5, the younger sister of patient 4, attended our clinic alongside her sister. She was clearly more mildly affected. She presented at 3 y 6 mo with developmental delay; she had never had any seizures. She started walking at 3 y, and 6 months later could take ten steps and had ten words. Although better than her sister, she is significantly delayed. She has never suffered regression. She was hypotonic with normal reflexes, joint laxity, very flat feet and a broad-based gait. She had hypermetropia, astigmatism and a right convergent squint. She is microcephalic and MRI at 2 y demonstrated delayed temporal pole myelination, prominent cerebellar folia and a small splenium. Absence seizures were detected at 7y.

Investigations showed a mildly elevated ammonia in patient 4 (56; ref: < 40 μmol/L) and some amino acid abnormalities (see below). Patient 4 had a muscle biopsy, which showed increased lipid, increased fibre size variation (scattered small fibres) and mild myopathic features. Respiratory chain complexes were normal.

#### Patient 6

Patient 6 presented at 1 mo with prolonged jaundice and delayed feeding. Investigations were unremarkable and the jaundice resolved. At 6 wks, she developed focal seizures and asymmetric clonic episodes and an ictal EEG showed a right parietal lobe origin. The semiology subsequently changed with clusters of infantile spasms and multifocal discharges without hypsarrhythmia. The interictal EEG then deteriorated to a semi-discontinuous pattern with high amplitude multifocal discharges. Seizures were resistant to phenobarbitone, vigabatrin, prednisolone, carbamazepine, levetiracetam and pyridoxal 5’-phosphate. At 10 mo, she is developmentally impaired with hypotonia, poor head control, delayed feeding and extremely delayed visual development. She has no visual fixation, smiling, cooing or motor control, although she feeds orally without choking. MRI was normal.

### Amino acid abnormalities

The index patients (patients 1, 4 and 6) had elevated plasma proline on all occasions tested (*n* = 11, 2 and 2 respectively) and proline was elevated in a recent sample from patient 2 (Supplementary Table 2). Glutamate was elevated in >50% of the samples from each patient. However, patients 3 and 5 had normal plasma proline. In patient 1, amino acids were assayed after a 6-hour fast and then post-prandially. Only proline was elevated in the fasting sample, but in post-prandial samples, several amino acids were elevated: leucine, isoleucine, valine, alanine, tyrosine, lysine and arginine. Random samples from patient 4 showed variable elevations of isoleucine, valine, alanine, lysine and arginine. Plasma amino acids from patients 2, 3 and 6 showed mild elevations of some of these amino acids plus glutamate and ornithine. A random plasma sample from patient 5 was normal. It should be stressed that most of the plasma samples were taken in a routine clinic setting and so values for glutamine and glutamate should be treated with some caution (spontaneous hydrolysis) as should values of arginine and ornithine (red cell arginase activity).

Urinary amino acid analysis showed raised glutamate in patients 1 and 5 and raised proline in patients 1, 5 and 6 (Supplementary Table 3). Patient 1 also had elevated CSF proline and patients 1 and 5 had a low CSF glutamate (Supplementary Table 4).

### Fibroblast vacuolation

**Fig. 1:**
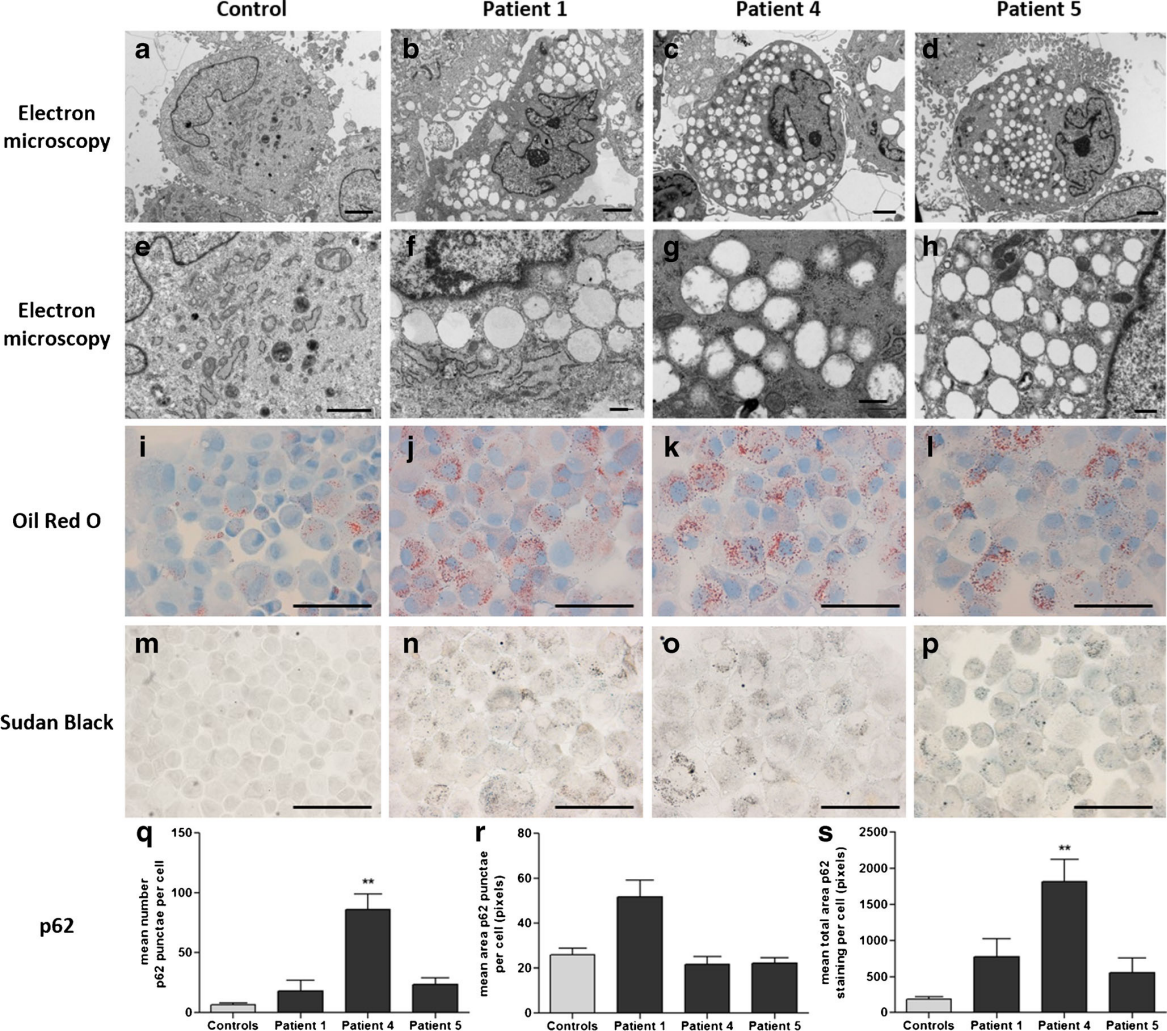
Structural and ultrastructural examinations of SLC25A22-deficient fibroblasts. Fibroblast cell culture from control (**a**, **e**, **i**, **m**, **q**), patient 1 (**b**, **f**, **j**, **n**, **r**), patient 4 (**c**, **g**, **k**, **o**, **s**) and patient 5 (**d**, **h**, **l**, **p**, **t**). Ultrastructural examination by electron microscopy revealed widespread, almost exclusively empty vacuoles in all patients (**a**-**h**). Patients showed excess accumulation of lipid (**i**-**l**, Oil Red O; **m**-**p**, Sudan Black). Immunofluorescence of the autophagy marker p62 revealed increases in number and area of p62 punctae in patient cells, indicating a possible increase in autophagy or mitophagy due to mitochondrial dysfunction. Four representative images of p62 immunofluorescence per case were analysed using CellProfiler 2.1.1. All patient cells showed an increase in the mean number of p62 punctae per cell, which was significant in patient 4 (*p* = 0.002) (**q**). The mean area of p62 punctae in patient 1 was also increased compared to controls (**r**). Taken together, the mean total area of p62 staining per cell is increased in all patients compared to controls and significantly in patient 4 (*p* = 0.002) (**s**). Scale bars: **a–e**: 2 μm; **f–h**: 500 nm; **i–p**: 100 μm; **q–t**: 50 μm

EM of fibroblasts from three patients showed extensive vacuolation with empty, single membrane-bound vacuoles in all. Tinctorial stains showed punctate staining using Oil Red O and Sudan Black indicating neutral lipids and phospholipids, respectively. Luxol Fast Blue staining for sphingomyelin was negative. Acid phosphatase and LAMP2 immunostaining was comparable to controls indicating no evidence of lysosomal dysfunction. Immunofluorescence of p62 in patient fibroblasts demonstrated an increase in number and size of autophagosomes compared to controls (Fig. 1), however, a statistically significant increase was only seen in one patient (Fig. 1W).

### Whole exome sequencing (WES)

*SLC25A22* sequence changes were identified in four cases by WES and in two by prenatal Sanger sequencing. Patients 1–3 are homozygous for c.166A>C; p.Thr56Pro, patients 4 and 5 are homozygous for c.886G>A; p.Ala296Thr and patient 6 is compound heterozygous for c.235G>A; p.Glu79Lys and c.746T>A; p.Val249Glu. All mutations were predicted ‘damaging’ by SIFT and all, with the exception of p.Val249Glu (predicted ‘possibly damaging’), were predicted ‘probably damaging’ by PolyPhen-2. None had been reported in Ensembl, 1000 Genomes or Exome Variant Server databases. A restriction enzyme digest test confirmed segregation of the p.Thr56Pro mutation. Sanger sequencing confirmed segregation of p.Ala296Thr, and confirmed maternal inheritance of p.Glu79Lys and paternal inheritance of p.Val249Glu. No potentially pathogenic changes were identified in genes known to cause hyperprolinaemia (*PRODH* and *ALDH4A1)*.

In light of the EM findings, WES data was scrutinised for variants in lysosomal storage disorder (LSD) genes. In one of the families, a homozygous variant (c.340G>A; p.Val114Met) was identified in *SMPD1* (Niemann-Pick disease type A/B; reference sequence NM_000543.4). This variant has a minor allele frequency of < 0.01 (1000 Genomes) and is predicted ‘tolerated’ and ‘benign’ by SIFT and PolyPhen-2, respectively. Leucocyte sphingomyelinase activity was 0.43 nmol/hr/mg protein (ref: 0.86–2.8) — lower than in heterozygotes (0.7–1.3), but higher than in affected homozygotes (0.08–0.18), indicating that this variant is probably not pathogenic.

## Discussion

WES identified four novel mutations in *SLC25A22* in six children from three families. They affect highly conserved regions of SLC25A22 (Supplementary Fig. 1), one of two mitochondrial glutamate transporters; *SLC25A18* encodes the paralogous glutamate transporter sharing 63% sequence identity with *SLC25A22*. All mutated amino acids, except Val249, are also conserved in SLC25A18; all mutations are predicted damaging. Both *SLC25A18* and *SLC25A22* are ubiquitously expressed, with *SLC25A22* present in higher amounts in all tissues except brain. SLC25A22 has a higher K_m_ and V_max_ than SLC25A18 with respect to glutamate (Fiermonte et al 2002), suggesting that SLC25A22 may become active to cope with higher cytosolic concentrations of glutamate. We considered the possibility that polymorphisms in *SLC25A18* might be responsible for the variation of the clinical and biochemical features between siblings (patients 1–3 and 4&5 respectively), however, no polymorphisms in *SLC25A18* were found in the exome sequencing data of any of these patients.

**Fig. 2:**
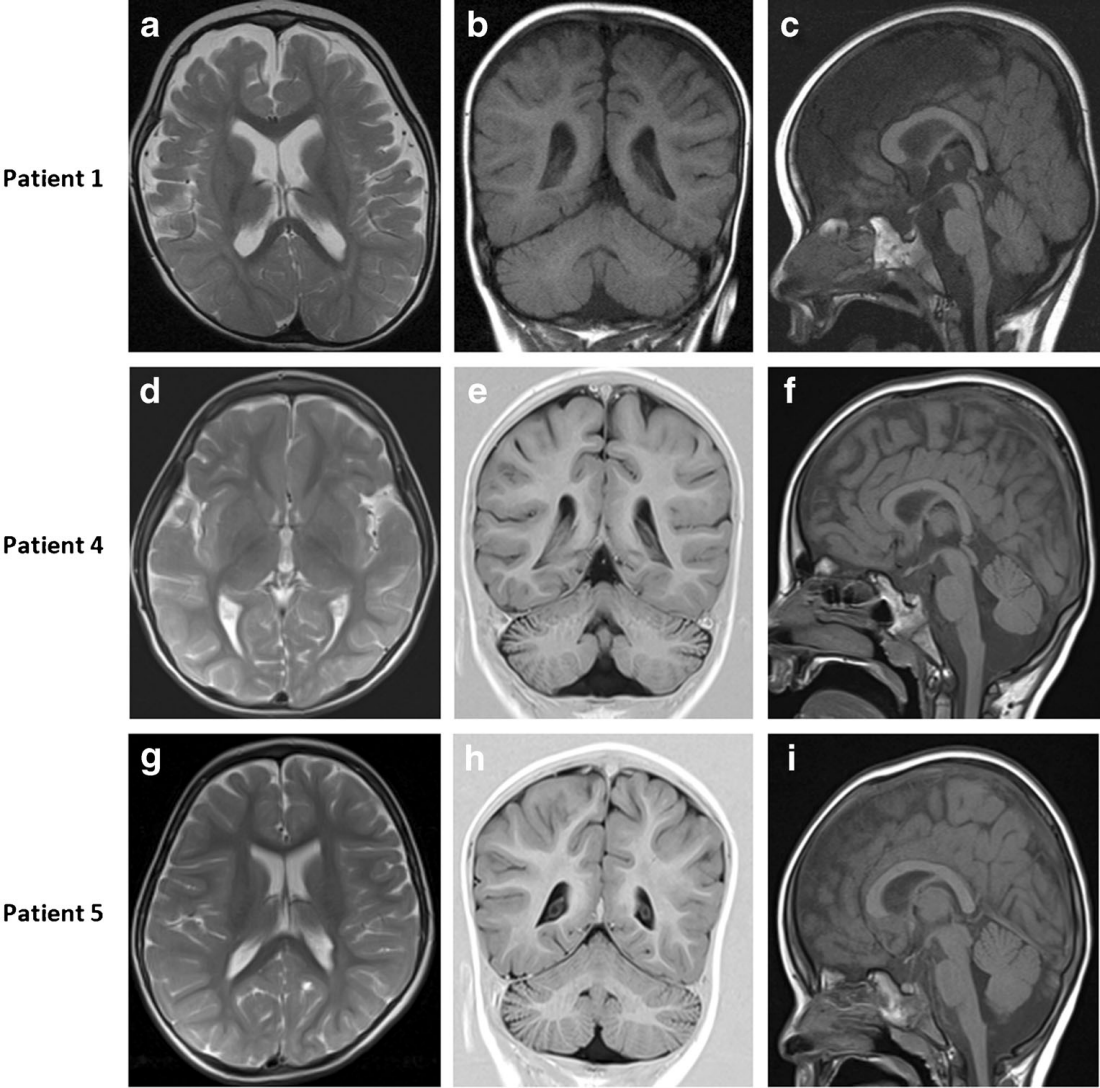
Brain magnetic resonance imaging of patients with SLC25A22 deficiency. (**a**, **d** and **g**) T2-weighted images. (**b**, **c**, **f** and **h**) T1-weighted images. (**e** and **h**) T1-weighted images with inversion recovery sequence. Patient 1 (2 y 1 m), patient 4 (6y 8 m) and patient 5 (2y 10 m). All patients show frontotemporal hypoplasia/atrophy (**a**, **d** and **g**) and prominence of cerebellar folia, consistent with cerebellar hypoplasia/atrophy (**b**, **e** and **h**). All patients also have a small splenium, with the splenium of the corpus callosum smaller than the genu and a small optic chiasma (smaller than the mammillary body) (**c**, **f** and **i**). Abnormal appearance of the insular cortex is also noted in patient 4 (**d**)

Despite harbouring novel mutations, patients 1, 2, 3, 4 and 6 presented like previous patients with marked hypotonia, onset of refractory seizures within the first 3 months of life, and developmental delay (Table 1, Supplementary Table 1). Patient 5, however, presented at 3 y 6 mo with developmental delay and only developed seizures at 7 y. MRI scans after age 2 y all showed a small splenium, prominent cerebellar folia and delayed temporal pole myelination as well as a small optic chiasma (consistent with post-retinal dysfunction) (Fig. 2). However, an MRI scan on patient 6 at 10 months was normal and patients 2 and 3 have not yet had a scan.

**Table 1:** Clinical phenotype and demographics of patients with SCL25A22 mutations

Clinical features & demographics	Incidence
Gender	Male; 8/15 (53%) Female; 7/15 (47%)
Country of origin	Afghanistan; 3/15 (20%) Syria; 2/15 (13%) Israel; 6/15 (40%) Algeria; 1/15 (7%) Saudi Arabia; 2/15 (13%) British/Finnish; 1/15 (7%)
Parental consanguinity	14/15 (93%)
Age at seizure onset	First week; 6/15 (40%) 1 week – 1 month; 3/15 (20%) 1–3 months; 5/15 (33%) 7 years; 1/15 (7%)
Seizure type	Neonatal/early infantile epileptic encephalopathy; 12/15 (80%) Migrating partial seizures of infancy; 2/15 (13%) Absence seizures in childhood; 1/15 (7%)
Response to AEDs	None; 6/12 (50%) Partial; 5/12 (42%) Yes; 1/12 (8%)
Distinct EEG features	Burst-suppression; 5/12 (42%) Multifocal; 5/12 (42%) Delta brush pattern; 1/12 (8%) Irregular slow wave activity; 1/12 (8%)
VEP	Abnormal; 5/8 (63%) Normal; 3/8 (37%)
ERG	Abnormal; 2/4 (50%) Normal; 2/4 (50%)
Other ocular findings described	No fixing/following; 2/13 (15%) No response to light; 5/13 (38%) Retinal pigmentation; 1/13 (8%) Hypermetropia, astigmatism, convergent squint; 1/13 (8%) Normal; 2/13 (15%)
Microcephaly	7/7 (100%)
MRI	Hypoplastic corpus callosum/splenium; 8/11 (72%) Cerebellar hypoplasia/prominent folia; 5/11 (45%) Delayed myelination; 4/11 (36%) Brain atrophy; 4/11 (36%) Subarachnoid enlargement; 2/11 (18%) White matter abnormalities; 2/11 (18%) Frontotemporal hypoplasia; 1/11 (9%) Normal; 1/11 (9%)
Biochemical findings	Persistently elevated plasma proline; 3/6 (50%) Intermittently elevated plasma proline; 1/6 (17%) Intermittently elevated plasma ornithine or arginine; 5/6 (83%) Vacuolated fibroblasts (mostly empty); 3/3 (100%)

*AEDs* antiepileptic drugs, *ERG* electroretinography, *VEP* visual evoked potential

**Fig. 3:**
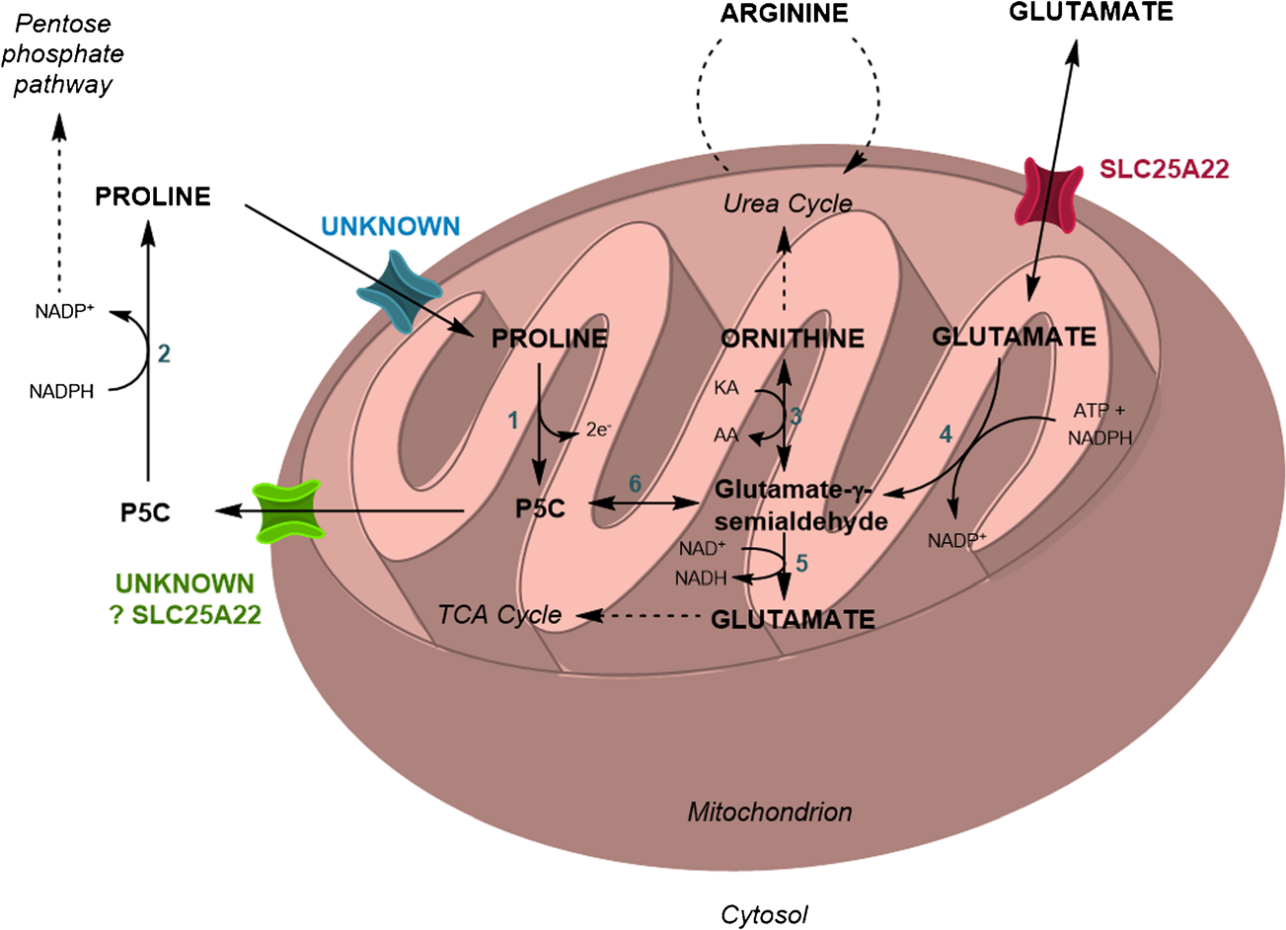
Metabolism of proline and proposed additional transporter function of SLC25A22. Pathway showing the synthesis, degradation and interconversions of proline, P5C and related compounds within the mitochondria. 1, proline dehydrogenase; 2, P5C reductase; 3, ornithine δ-aminotransferase; 4, P5C synthase; 5, P5C dehydrogenase; 6, non-enzymatic; KA, keto-acid; AA, amino acid

SLC25A22 deficiency has been considered to cause an early-onset seizure disorder with no associated biochemical abnormalities. However, in this series, one patient was referred with hyperprolinaemia and in another three it was detected on first line investigations. The metabolic pathways for catabolism, cycling and synthesis of proline are shown in Fig. 3. Proline enters mitochondria from the cytosol and proline dehydrogenase converts proline to pyrroline-5-carboxylate (P5C). In bacteria, the reducing equivalents are transferred to ubiquinone to form ubiquinol (Natarajan and Becker 2012). P5C can then be transported out of the mitochondrion or further metabolised. The P5C efflux transporter is unknown (Miller et al 2009). However, cytosolic P5C can be converted back to proline by P5C reductase using reducing equivalents from NADPH, thus completing the proline/P5C shuttle, the net result of which is transfer of reducing equivalents from cytosolic NADPH to the mitochondrial respiratory chain.

Alternatively, P5C in mitochondria can undergo non-enzymatic hydrolysis to generate glutamate-γ-semialdehyde (GSA). GSA can be further metabolised to either glutamate or ornithine and arginine. All the intramitochondrial metabolic interconversions can influence plasma amino acid concentrations, as exemplified by the abnormalities characteristic of deficiencies of proline dehydrogenase, P5C dehydrogenase, P5C synthase and ornithine δ-aminotransferase (Baumgartner et al 2005).

In many cells, glutamate is synthesised from 2-oxoglutarate produced in the Krebs cycle and the net flux of glutamate and P5C is likely to be from mitochondrion to cytosol. In patients with SLC25A22 deficiency, defective mitochondrial export of glutamate or P5C could lead to high intramitochondrial concentrations and, as a consequence, reduced catabolism of proline and increased synthesis of ornithine and arginine. We suggest that the fact that 4/6 (67%) patients had persistently or intermittently elevated plasma proline and 5/6 (83%) had elevated plasma arginine and/or ornithine on at least one occasion supports the hypothesis that export of glutamate and/or P5C from the mitochondrion is disturbed. This hypothesis requires that an important function of SLC25A22 is to transport glutamate and/or GSA/P5C out of the mitochondria in addition to acting as a glutamate importer. Bidirectional transport of glutamate is supported by studies in liposomes (Fiermonte et al 2002).

In liver cells the net flux of glutamate through the SLC25A22 transporter is probably from cytosol to mitochondria in the post prandial period. Post-prandial plasma samples from patient 1 showed elevated leucine, isoleucine, valine, alanine, tyrosine, lysine and threonine (Supplementary Table 2 and Fig. 2); some of these amino acids were elevated in random samples from other patients. In the fed state, amino acids are the preferred fuel of the liver. The influx from the portal vein leads to high concentrations in the cytosol of hepatocytes. Leucine, isoleucine, valine, alanine and tyrosine all undergo (reversible) transamination with 2-oxoglutarate to produce glutamate and the glutamate needs to be transported into the mitochondrion for deamination. SLC25A22 likely fulfils this role. In the absence of SLC25A22, high cytosolic levels of glutamate will impair transamination and hence the plasma concentrations of the amino acids are higher than normal in the post-prandial period. It is possible that a standardised protein load will prove to be a better marker of SLC25A22 deficiency than a random plasma proline but further work will be needed to develop this.

Changes in glutamate concentration in mitochondria or in the cytosol will be associated with corresponding changes in 2-oxoglutarate because of the presence of transaminases in both sites. Changes in glutamate and 2-oxoglutarate can be expected to affect the oxoglutarate/malate and aspartate/glutamate transporters involved in the malate shuttle. These could be reflected by abnormalities of plasma aspartate and of urinary excretion of organic acids such as malate and 2-oxoglutarate. However, the effects are likely to be opposite in situations where the net flux through SLC25A22 is cytosol > mitochondrion and mitochondrion > cytosol and these opposite effects may cancel one another out.

As indicated in Supplementary Table 3, plasma aspartate levels were all normal except the one measured in patient 1 at 2 y 3 mo (32 μmol/L; ref 0–14). Interestingly this was also the sample with the highest proline level. No obvious abnormalities of organic acids were detected.

Patient 1 had a low white cell ubiquinone. Disruption of the proline/P5C shuttle may lead to reduced flux through proline dehydrogenase. The electron acceptor for proline dehydrogenase in man is unknown, but in bacteria is ubiquinone (Natarajan and Becker 2012). Perhaps a reduced proline flux somehow leads to increased losses of ubiquinone. Unfortunately, ubiquinone treatment of patients 2 and 3 did not prevent the onset of seizures.

EM of fibroblasts demonstrated widespread vacuolation, with Oil Red O and Sudan Black staining indicating the presence of neutral lipids and phospholipids (Fig. 1). Lipid synthesis can be driven by increased cytosolic NADPH and citrate. In both proliferating and quiescent fibroblasts the pentose phosphate pathway produces cytosolic NADPH (Lemons et al 2010). Impairment of the proline/P5C shuttle could lead to increased cytosolic NADPH/NADP^+^. Increased intra-mitochondrial glutamate is likely to lead to excessive Krebs cycle anaplerosis (or acetyl-CoA production via malic enzymes) and hence excess mitochondrial citrate production leading to export of citrate for lipogenesis. Furthermore, when quiescent fibroblasts are fed [U-^13^C]-glutamine the label appears in [5x^13^C]-citrate indicating some conversion as follows: glutamine > glutamate > 2-oxoglutarate > citrate. Export of citrate and build-up of cytosolic NADPH is one of the postulated mechanisms for cytosolic lipid accumulation in respiratory chain disorders. Our hypothesis of impairment of the proline/P5C shuttle in SLC25A22 deficiency requires that the protein transports P5C or GSA out of the mitochondrion. The transporter responsible for this function is not known; SLC25A22 is a possible candidate given the similarity of GSA to glutamate.

Although the vacuoles did not contain organelle debris, we investigated whether SLC25A22 deficiency may be inducing autophagy. Autophagy can be induced by changes in amino acid levels (Dodson et al 2013). Staining for p62 did show an increase in number and size of autophagosomes (Fig. 1), statistically significant in one patient (Fig. 1W).

Lipid-containing vacuoles are seen in some LSDs. Therefore, WES data was scrutinised for variants in LSD genes and a homozygous rare variant in *SMPD1* (p.Val113Met) was identified in one family. Sphingomyelinase activity was below the reference range but above that of affected individuals. Since vacuolation was similar in all patients and there was no histochemical evidence of sphingomyelin accumulation, it was concluded that this variant was not pathogenic. Thus, we conclude that none of the patients have a second genetic defect leading to lipid accumulation.

Patient 1 had a low CSF pyridoxal 5’-phosphate (PLP). In hyperprolinaemia type II (HP2), accumulation of P5C leads to inactivation of PLP. If, as we have proposed, levels of P5C are high in the mitochondrion then PLP might be inactivated in this site and perhaps, as in HP2, this inactivation may contribute to seizures.

In conclusion, mutations in *SLC25A22* cause developmental delay with late-onset seizures as well as neonatal-onset seizures. Biochemical abnormalities can include hyperprolinaemia, and lipid accumulation, features that may be useful markers. Increased cytosolic lipid synthesis could indicate increased cytosolic NADPH and this, alongside hyperprolinaemia, suggests impairment of the proline/P5C shuttle. Thus, we hypothesise that SLC25A22 may transport pyrroline-5-carboxylate/glutamate-γ-semialdehyde as well as glutamate.

## Electronic supplementary material

Below is the link to the electronic supplementary material.

Supplementary Methods (DOCX 16 kb)

Supplementary Figure 1 (DOCX 2921 kb)

Supplementary Figure 2 (DOCX 148 kb)

Supplementary Table 1 (DOCX 20 kb)

Supplementary Table 2 (DOCX 19 kb)

Supplementary Table 3 (DOCX 15 kb)

Supplementary Table 4 (DOCX 13 kb)

## Abbreviations

GSA: Glutamate-γ-semialdehyde
EEG: Electroencephalogram
HP2: Hyperprolinaemia type II
LAMP2: Lysosome-associated membrane protein 2
LSD: Lysosomal storage disease
MRI: Magnetic resonance imaging
NAD^+^: Nicotinamide adenine dinucleotide (oxidised form)
NADPH: Nicotinamide adenine dinucleotide phosphate (reduced form)
P5C: Pyrroline-5-carboxylate
p62: Nucleoporin 62
PLP: Pyridoxal 5’-phosphate
PND: Prenatal diagnosis
SLC25A22: Solute carrier family 25 (mitochondrial carrier: glutamate) member 22
VEP: Visual evoked potential
WES: Whole exome sequencing
