# Supplementary material for: Mutations in SLC25A22: hyperprolinaemia, vacuolated fibroblasts and presentation with developmental delay
Source: J Inherit Metab Dis. 2017 Mar 2;40(3):385–94. doi: 10.1007/s10545-017-0025-7 (PMC5393281; doi:10.1007/s10545-017-0025-7)
Supplement: Supplementary file 1 — (DOCX 16 kb) [file 10545_2017_25_MOESM1_ESM.docx]

**Supplementary Methods**

***SLC25A22* PCR reaction mix, PCR conditions, primer sequences and multiple sequence alignment details.**

PCR reaction mix:

| Component | Volume (µL) |
| --- | --- |
| dH_2_O | 14.30 |
| Buffer (10X) | 2.5 |
| MgCl_2_ (25 mM) | 0.75 |
| dNTPs (200 mM) | 0.75 |
| Forward Primer (10 µM) | 1.25 |
| Reverse Primer (10 µM) | 1.25 |
| 5% DMSO | 1.25 |
| Taq (BioTaq DNA polymerase [5 U/µl] | 0.2 |
| DNA [75 ng/µl] | 1 |
| Total | 25 |

PCR conditions:

| Step | Conditions |
| --- | --- |
| 1 | 96°C for 5 minutes |
| 2 | 96°C for 30 seconds |
| 3 | T_m_°C for 30 seconds  *(66°C for p.Thr56Pro and 68°C for p.Ala296Thr)* |
| 4 | 72°C for 30 seconds |
| 5 | Repeat steps 2-4 34 times for a total of 35 cycles |
| 6 | 72°C for 10 minutes |

Primers used for SLC25A22 sequencing

**Patients 1 – 3 (c.166A>C; p.Thr56Pro)**

Forward (5’ – 3’): TGAGGACTTGGCCTCTTCTATC

Reverse (5’ – 3’): ACGTCCACGCTCACACAC

**Patients 4 and 5 (c.886G>A; p.Ala296Thr)**

Forward (5’ – 3’): CTCTGGGATCCTGGACTGTG

Reverse (5’ – 3’): CTATGTGGACCCTGCACCC

SLC25A22 sequence alignment details

| Species | Ensembl transcript ID | UniProt | CCDS | RefSeq |
| --- | --- | --- | --- | --- |
| Human | ENST00000320230 | Q9H936 | CCDS7715 | NM_024698 |
| Gorilla | ENSGGOT00000010810 | G3R5C9 | - | - |
| Mouse | ENSMUST00000019226 | Q9D6M3 | CCDS22013 | NM_026646 |
| Zebrafish | ENSDART00000103385 | Q7ZTT6 | - | NM_213408 |
| Kangaroo rat | ENSDORT00000016456 | - | - | - |
| Cat | ENSFCAT00000004818 | M3W2X0 | - | - |
| Horse | ENSECAT00000012473 | F6VF01 | - | - |
| Pig | ENSSSCT00000014039 | F1RYY8 | - | - |
| Wallaby | ENSMEUT00000000836 | - | - | - |
| Coelacanth | ENSLACT00000024911 | M3XGX3 | - | - |

**Nucleoporin (p62) immunofluorescence and quantification**

Immunofluorescence was carried out using the method of Yasin *et al*. (2013). Briefly, glass coverslips were treated with 1N hydrochloric acid and coated with 5 μg/ml poly-D-lysine. Fibroblasts were seeded on the coverslips at a density of 2.5 x 10^4^ cells/mL, cultured to confluence for 24 hours, fixed with 4% paraformaldehyde for 15 mins at room temperature and washed three times with phosphate buffered saline (PBS). Cells were blocked using blocking solution (0.1% Triton X-100, 10% Sheep serum in PBS) for 1 hour at room temperature. Autophagosomes were stained by incubating cells with p62 primary antibody (1:50, BD Biosciences) at 4°C overnight. Cells were washed twice with PBS and then incubated with Alexa Fluor 610 secondary antibody (1:240, Molecular Probes) in the dark for 1 hour at room temperature. Slides were washed twice in PBS and mounted in Vectashield aqueous mounting medium containing 4′, 6-diamidino-2-phenylindole/DAPI (Vector). Slides were visualised and images captured using a Leica DMLB fluorescent microscope.

Cells were counted by quantitation of nuclear DAPI staining using minimum and maximum diameter settings of 70 and 300 pixels, respectively. The threshold correction factor was set to 1 using the Otsu Global thresholding method in two-class mode. Staining of p62 punctae was also counted using a diameter range of 1 to 30 pixels and a threshold correction factor of 2.2. The Otsu Global thresholding method in three-class mode was used with the middle intensity assigned to the background to avoid counting background as cells. All other pipeline settings were kept as default.

**Electron Microscopy**

Cells were fixed in 2.5% glutaraldehyde with 0.1M sodium cacodylate (pH 7.2) for 24h and postfixed in 1% osmium tetroxide, dehydrated in graded ethanol, processed through propylene oxide and embedded in Agar 100 epoxy resin. Ultrathin 90 nm sections were cut with a diamond knife (Leica Ultracut UCT Ultramicrotome), placed on copper grids, stained with uranyl acetate and lead citrate (Yasin et al 2013) and examined with a JEOL 1400 transmission electron microscope.
