# Supplementary material for: Mutations in SLC25A22: hyperprolinaemia, vacuolated fibroblasts and presentation with developmental delay
Source: J Inherit Metab Dis. 2017 Mar 2;40(3):385–94. doi: 10.1007/s10545-017-0025-7 (PMC5393281; doi:10.1007/s10545-017-0025-7)
Supplement: Supplementary file 2 — (DOCX 2921 kb) [file 10545_2017_25_MOESM2_ESM.docx]

**
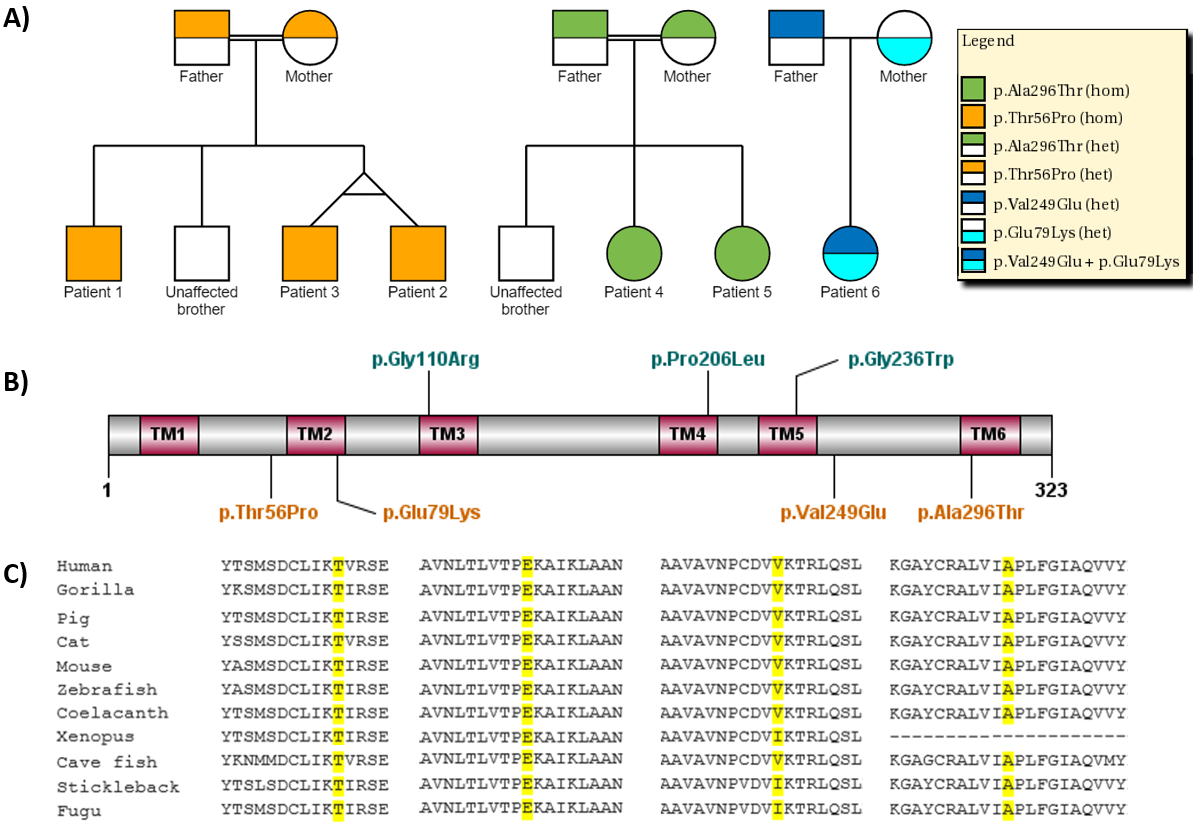
**

**Supplementary Figure 1:** **Position and conservation of novel *SLC25A22* mutations.** **(A)** Pedigrees of family 1, 2 and 3 depicting the relationship between the affected patients. **(B)** Schematic of the *SLC25A22* gene showing the positions of the mutations described in the literature (blue) and the mutations identified in our families (red). **(C)** Multiple sequence alignment of the SLC25A22 protein across species generated using ClustalW2 (Thompson *et al.*, 1994) illustrating the conservation of the sites of the p.Thr56Pro, p.Glu79Lys, p.Val249Glu and p.Ala296Thr mutations, respectively. Details of all aligned proteins are given in Supplementary Material 7.
