# Supplementary material for: Mutations in SLC25A22: hyperprolinaemia, vacuolated fibroblasts and presentation with developmental delay
Source: J Inherit Metab Dis. 2017 Mar 2;40(3):385–94. doi: 10.1007/s10545-017-0025-7 (PMC5393281; doi:10.1007/s10545-017-0025-7)
Supplement: Supplementary file 3 — (DOCX 148 kb) [file 10545_2017_25_MOESM3_ESM.docx]

**
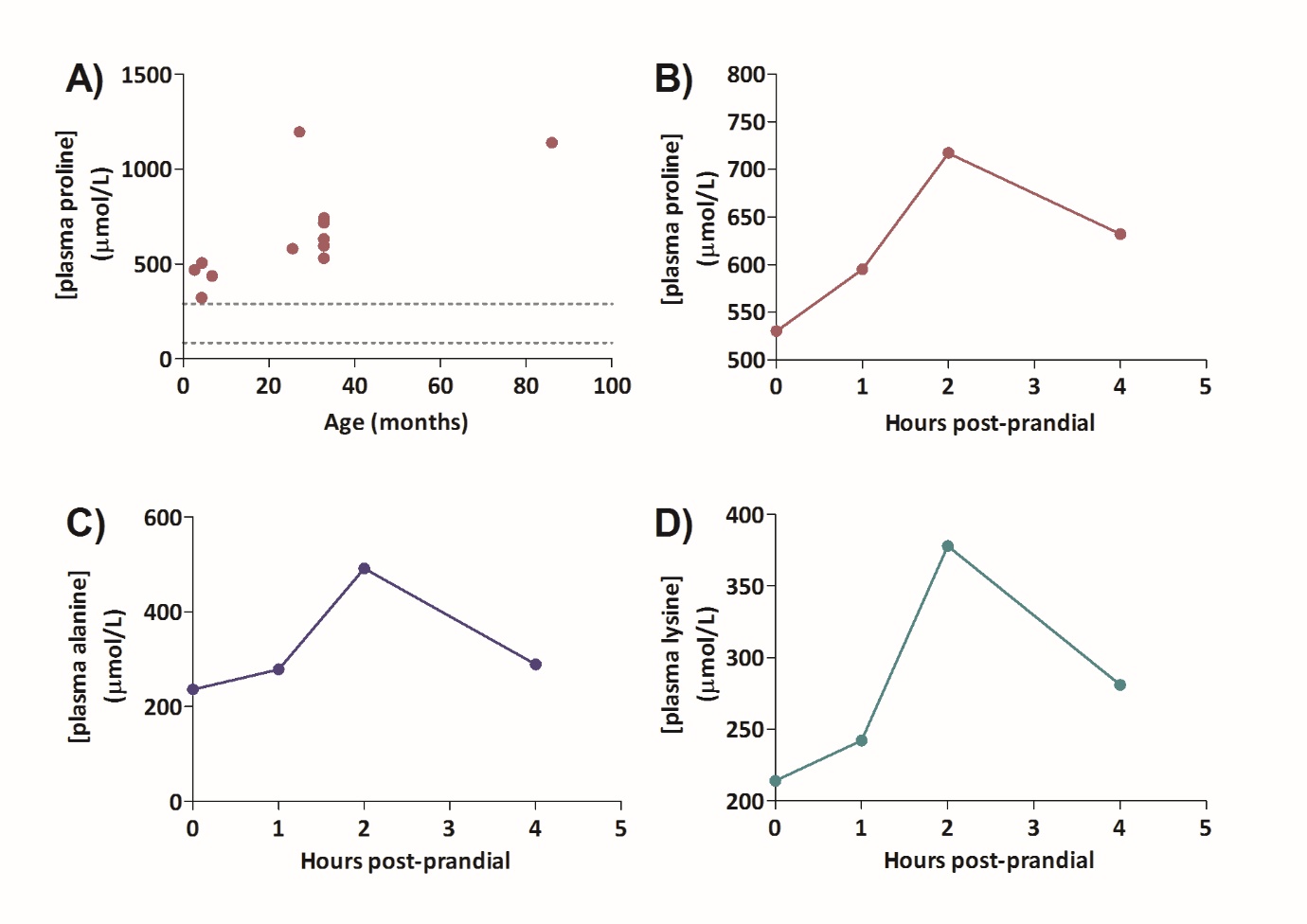
Supplementary Figure 2:** **Variation in plasma amino acids with age and post-prandial. (A)** Plasma proline concentrations in patient 1 over time. Multiple data points at 32.8 months represent pre- and post-prandial samples. Dotted lines represent reference ranges. **(B, C and D)** Plasma concentrations in patient 1 in response to feeding showing proline (ref: 85 – 290 µmol/L), alanine (ref: 150 – 450 µmol/L) and lysine (ref: 100 – 300 µmol/L), respectively. Samples were taken after a six hour fast and 1, 2, and 4 hours after eating.
