# Supplementary material for: Mutations in SLC25A22: hyperprolinaemia, vacuolated fibroblasts and presentation with developmental delay
Source: J Inherit Metab Dis. 2017 Mar 2;40(3):385–94. doi: 10.1007/s10545-017-0025-7 (PMC5393281; doi:10.1007/s10545-017-0025-7)
Supplement: Supplementary file 4 — (DOCX 20 kb) [file 10545_2017_25_MOESM4_ESM.docx]

**Supplementary Table 1: Phenotypic comparison of our six patients compared to those described in the literature with mutations in *SLC25A22*.**

|  | Patient 1 | Patient 2 | Patient 3 | Patient 4 | Patient 5 | Patient 6 | Molinari et al. (2005)  n=4 | Molinari et al. (2009)  n=1 | Poduri et al. (2013)  n=2 | Cohen et al. (2014)  n=2 |
| --- | --- | --- | --- | --- | --- | --- | --- | --- | --- | --- |
| Patient demographics | | | | | | | | | |  |
| Country of origin | Afghanistan | Afghanistan | Afghanistan | Syria | Syria | Britain/Finland | Israel | Algeria | Saudi Arabia | Israel |
| Consanguineous | + | + | + | + | + | - | +/+/+/+ | + | +/+ | +/+ |
| Mutations | c.166A>C; p.Thr56Pro | c.166A>C; p.Thr56Pro | c.166A>C; p.Thr56Pro | c.886G>A; p.Ala296Thr | c.886G>A; p.Ala296Thr | c.235G>A; p.Glu79Lys  c.746T>A; p.Val249Glu | c.617C>T;  p.Pro206Leu | c.706G>T; p.Gly236Trp | c.328G>C; p.Gly110Arg | c.617C>T;  p.Pro206Leu |
| Features at presentation | | | | | | | | | | |
| Refractory seizures | + | + | + | + | - | + | +/+/+/+ | + | +/+ | +/+ |
| Hypotonia | + | + | + | + | + | + | +/+/+/+ | + | +/+ | +/+ |
| Developmental delay | - | - | - | - | + | - | -/-/-/- | - | -/- | -/- |
| Age at seizure onset |  |  |  |  |  |  |  |  |  |  |
| < 48 hours | - | - | - | - | - | - | +/+/+/+ | - | -/- | -/- |
| 4 hours – 1 week | - | - | - | - | - | - | -/-/-/- | + | +/- | -/- |
| 2 weeks | - | - | - | - | - | - | -/-/-/- | - | -/+ | +/- |
| 2 weeks – 4 weeks | - | - | - | + | - | - | -/-/-/- | - | -/- | -/+ |
| 6 weeks | + | - | - | - | - | + | -/-/-/- | - | -/- | -/- |
| 10 weeks | - | + | + | - | - | - | -/-/-/- | - | -/- | -/- |
| 7 years | - | - | - | - | + | - | -/-/-/- | - | -/- | -/- |
| EEG findings | | | | | | | | | | |
| Abnormal EEG | + | + | + | + | nd | + | +/+/nd/nd | + | +/+ | +/+ |
| Burst-suppression on EEG | - | - | - | - | nd | - | +/+/nd/nd | + | -/- | +/+ |
| Multifocal EEG abnormalities | - | + | + | - | nd | + | -/-/nd/nd | - | -/- | -/- |
| Symmetrical diffuse irregular slow wave activity | - | - | - | + | nd | - | -/-/nd/nd | - | -/- | -/- |
| Hypsarrhythmia | - | - | - | - | nd | - | -/-/nd/nd | + | -/- | -/- |
| Delta brush pattern | - | - | - | - | nd | - | -/-/nd/nd | - | +/- | -/- |
| Positive spikes and high-voltage focal spikes | - | - | - | - | nd | - | -/-/nd/nd | - | +/+ | -/- |
| Ocular abnormalities | | | | | | | | | | |
| No response to light | + | - | - | - | - | - | -/-/nd/nd | - | -/- | +/+ |
| No fixing and following | - | + | + | - | - | + | -/-/nd/nd | - | -/- | -/- |
| Abnormal VEP | + | - | - | - | - | - | +/+/nd/nd | + | +/- | -/- |
| Abnormal ERG | - | - | - | - | - | - | +/+/nd/nd | + | -/- | -/- |
| Hypermetropia, an astigmatism and right convergent squint | - | - | - | - | + | - | - | - | - | - |
| Retinal pigmentation | - | - | - | - | - | - | - | - | - | +/- |
| MRI features | | | | | | | | | | |
| Hypoplastic corpus callosum/splenium | + | nd | nd | + | + | - | +/-/nd/nd | + | +/- | +/+ |
| Cerebellar hypoplastic/prominent cerebellar folia | + | nd | nd | + | + | - | +/-/nd/nd | + | -/- | -/- |
| Delayed myelination | + | nd | nd | + | + | - | -/-/nd/nd | + | +/- | -/- |
| Frontotemporal hypoplasia | + | nd | nd | - |  |  | -/-/nd/nd | - | -/- | -/- |
| Generalised brain atrophy | - | nd | nd | - | - | - | +/+/nd/nd | - | -/- | +/+ |
| Subarachnoid enlargement | - | nd | nd | - | - | - | +/-/nd/nd | - | -/- | +/- |
| Symmetrical signal abnormalities of the insular cortex bilaterally and adjacent capsular white matter | - | nd | nd | + | - | - | -/-/nd/nd | - | -/- | -/- |

ERG, electroretinography; nd, not done; VEP, visual evoked potential.
