# Supplementary material for: Mutations in SLC25A22: hyperprolinaemia, vacuolated fibroblasts and presentation with developmental delay
Source: J Inherit Metab Dis. 2017 Mar 2;40(3):385–94. doi: 10.1007/s10545-017-0025-7 (PMC5393281; doi:10.1007/s10545-017-0025-7)
Supplement: Supplementary file 5 — (DOCX 19 kb) [file 10545_2017_25_MOESM5_ESM.docx]

**Supplementary Table 2:** **Plasma amino acids by high-performance liquid chromatography (HPLC) analysis measured in all patients.**

|  | Patient 1 (µmol/L) | | | | | | | | | | | Reference range (µmol/L) |
| --- | --- | --- | --- | --- | --- | --- | --- | --- | --- | --- | --- | --- |
|  | **2 months 19 days** | **4 months 10 days** | **4 months 11 days** | **6 months 23 days** | **2 years 3 months 4 days** | **2 years 8 months 25 days** | **2 years 8 months 25 days** | **2 years 8 months 25 days** | **2 years 8 months 25 days** | **2 years 8 months 26 days** | **7 years 2 months 18 days** |  |
| Sample time | **Random** | **Random** | **Random** | **Random** | **Random** | **6hr-fast** | **1hr pp** | **2hrs pp** | **4hrs pp** | **Random** | **Random** |  |
| Glycine | 221 | 226 | 372 | 239 | 434 | 179 | 182 | 205 | 178 | 200 | 363 | 100 – 330 |
| Serine | 197 | 203 | 260 | 182 | 629 | 189 | 201 | 237 | 212 | 232 | 278 | 90 – 290 |
| Threonine | 135 | 100 | 168 | 143 | 420 | 161 | 187 | 242 | 199 | 236 | 287 | 70 – 220 |
| Proline | 468 | 322 | 506 | 437 | 1195 | 530 | 595 | 717 | 632 | 743 | 1139 | 85 – 290 |
| Leucine | 119 | 91 | 79 | 132 | 265 | 151 | 159 | 255 | 179 | 223 | 170 | 65 – 220 |
| Isoleucine | 71 | 59 | 54 | 71 | 157 | 90 | 89 | 138 | 95 | 115 | 106 | 26 – 100 |
| Valine | 181 | 144 | 162 | 214 | 480 | 294 | 297 | 393 | 319 | 365 | 314 | 90 – 300 |
| Alanine | 329 | 306 | 535 | 329 | 913 | 236 | 278 | 491 | 289 | 407 | 758 | 150 – 450 |
| Glutamine | 914 | 659 | 745 | 991 | 737 | 642 | 656 | 757 | 671 | 759 | 796 | 480 – 800 |
| Arginine | 101 | 53 | 72 | 112 | 228 | 91 | 97 | 142 | 139 | 141 | 126 | 40 – 120 |
| Ornithine | 138 | 58 | 65 | 102 | 143 | 78 | 85 | 89 | 77 | 105 | 118 | 25 – 120 |
| Lysine | 233 | 125 | 122 | 211 | 470 | 214 | 242 | 378 | 281 | 351 | 359 | 100 – 300 |
| Methionine | 22 | 22 | 31 | 20 | 48 | 19 | 25 | 41 | 26 | 34 | 34 | 10 – 60 |
| Taurine | 109 | 44 | 40 | 94 | 71 | 48 | 136 | 54 | 35 | 49 | 91 | 40 – 140 |
| Phenylalanine | 53 | 37 | 42 | 40 | 100 | 45 | 47 | 74 | 54 | 63 | 57 | 35 – 100 |
| Tyrosine | 73 | 74 | 55 | 64 | 146 | 54 | 65 | 132 | 84 | 113 | 100 | 30 – 120 |
| Tryptophan | 34 | 28 | 26 | 28 | 71 | 36 | 31 | 55 | 34 | 42 | 31 | 30 – 80 |
| Histidine | 77 | 66 | 72 | 80 | 168 | 72 | 74 | 94 | 74 | 87 | 96 | 30 – 150 |
| Asparagine | 56 | 47 | 63 | 51 | 124 | 55 | 59 | 92 | 66 | 79 | 71 | - |
| Aspartate | 9 | 4 | 9 | 4 | 32 | 2 | 3 | 3 | 2 | 3 | 5 | 0 - 14 |
| Glutamate | 86 | 147 | 327 | 146 | 138 | 71 | 94 | 54 | 64 | 80 | 147 | 25 – 130 |

|  | Patient 2  (µmol/L) | | Patient 3  (µmol/L) | | Patient 4 (µmol/L) | | Patient 5 (µmol/L) | Reference range (µmol/L) | Patient 6 (µmol/L) | | Reference range (µmol/L) |
| --- | --- | --- | --- | --- | --- | --- | --- | --- | --- | --- | --- |
|  | **3 months 16 days** | **11 months** | **3 months 16 days** | **11 months** | **7 years 6 months 22 days** | **8 years 0 months 20 days** | **4 years 6 months 25 days** |  | **1 month** | **10 months** |  |
| Sample time | **Random** | **Random** | **Random** | **Random** | **Random** | **Random** | **Random** |  | **Random** | **Random** |  |
| Glycine | 265 | 164 | 216 | 170 | 385 | 427 | 222 | 100 – 330 | 209 | 270 | 160 – 304 |
| Serine | 249 | 168 | 187 | 135 | 275 | 210 | 166 | 90 – 290 | 218 | 165 | 70 – 178 |
| Threonine | 250 | 116 | 185 | 94 | 185 | 150 | 134 | 70 – 220 | 205 | 268 | 75 – 203 |
| Proline | 261 | 418 | 197 | 274 | 467 | 368 | 115 | 85 – 290 | 493 | 842 | 70 – 300 |
| Leucine | 144 | 192 | 108 | 98 | 205 | 82 | 103 | 65 – 220 | 181 | 205 | 85 – 169 |
| Isoleucine | 101 | 115 | 73 | 62 | 130 | 60 | 52 | 26 – 100 | 100 | 115 | 41 – 93 |
| Valine | 211 | 319 | 167 | 191 | 339 | 176 | 188 | 90 – 300 | 237 | 342 | 161 – 285 |
| Alanine | 582 | 328 | 321 | 285 | 716 | 820 | 230 | 150 – 450 | 467 | 814 | 155 – 537 |
| Glutamine | 848 | 614 | 764 | 493 | 851 | 711 | 757 | 480 – 800 | 1091 | 849 | 424 – 728 |
| Arginine | 188 | 166 | 159 | 135 | 129 | 115 | 88 | 40 – 120 | 156 | 183 | 49 – 129 |
| Ornithine | 132 | 60 | 118 | 43 | 142 | 107 | 53 | 25 – 120 | 228 | 135 | 21 – 77 |
| Lysine | 357 | 280 | 280 | 182 | 300 | 124 | 155 | 100 – 300 | 284 | 303 | 142 – 198 |
| Methionine | 40 | 32 | 30 | 21 | 40 | 18 | 18 | 10 – 60 | 38 | 38 | 17 – 37 |
| Taurine | 98 | 53 | 69 | 58 | 111 | 101 | 64 | 40 – 140 | 78 | 55 | 0 – 232 |
| Phenylalanine | 61 | 95 | 52 | 68 | 60 | 37 | 42 | 35 – 100 | 72 | 75 | 42 – 74 |
| Tyrosine | 113 | 106 | 86 | 63 | 125 | 57 | 46 | 30 – 120 | 104 | 68 | 40 – 94 |
| Tryptophan | 83 | 59 | 60 | 43 | 29 | 21 | 45 | 30 – 80 | * | * | * |
| Histidine | 86 | 87 | 71 | 70 | 104 | 83 | 80 | 30 – 150 | 103 | 85 | 65 – 105 |
| Asparagine | 113 | 81 | 81 | 59 | 78 | * | 53 | - | * | * | * |
| Aspartate | 9 | 8 | 7 | 5 | 5 | * | 5 | - | 13 | 7 | 0 – 14 |
| Glutamate | 150 | 139 | 117 | 53 | 100 | 149 | 95 | 25 – 130 | 116 | 94 | 8 – 64 |

Amino acid concentrations elevated above the reference range are shown in orange and those below the reference range are shown in blue.

pp = postprandial

* Amino acids were not quantified at this time. Amino acid concentrations in patient 6 were measured at a different centre, thus the reference ranges are different.
