# Supplementary material for: Mutations in SLC25A22: hyperprolinaemia, vacuolated fibroblasts and presentation with developmental delay
Source: J Inherit Metab Dis. 2017 Mar 2;40(3):385–94. doi: 10.1007/s10545-017-0025-7 (PMC5393281; doi:10.1007/s10545-017-0025-7)
Supplement: Supplementary file 6 — (DOCX 15 kb) [file 10545_2017_25_MOESM6_ESM.docx]

**Supplementary Table 3:** **Urine amino acids measured in patients 1 and 5 by ion exchange chromatography.**

|  | Patient 1 (µmol/mmol creatinine) | Reference range (µmol/mmol creatinine) | Patient 1 (µmol/mmol creatinine) | Reference range (µmol/mmol creatinine) | Patient 5 (µmol/mmol creatinine) | Reference range (µmol/mmol creatinine) | Patient 6 (µmol/mmol creatinine) | Reference range (µmol/mmol creatinine) |
| --- | --- | --- | --- | --- | --- | --- | --- | --- |
|  | **2 months 21 days** |  | **2 years 1 month 20 days** |  | **4 years 6 months 22 days** |  | **10 months 0 days** |  |
| Glycine | 1190 | 300 – 950 | 374 | 250 – 626 | 277 | 100 – 400 | 1326 | 40 – 616 |
| Serine | 316 | 25 – 95 | 19 | 20 – 100 | 67 | 20 – 50 | 8 | 30 – 191 |
| Threonine | 147 | 10 – 45 | 19 | 10 – 45 | 39 | 10 – 25 | 736 | 9 – 100 |
| Proline | 719 | 5 – 110 | 10 | 0 – 3 | 5 | 0 – 3 | 104 | 0 – 34 |
| Leucine | 17 | 5 – 20 | 2 | 3 – 10 | 9 | 3 – 10 | 4 | 3 – 21 |
| Isoleucine | 9 | 2 – 40 | <1 | 2 – 10 | 3 | 1 – 10 | 21 | 0 – 10 |
| Valine | 13 | 2 – 10 | <1 | 2 – 8 | 12 | 2 – 8 | 5 | 4 – 32 |
| Alanine | 382 | 30 – 130 | 68 | 30 – 80 | 60 | 20 – 80 | 1212 | 34 – 189 |
| Glutamine | 363 | 40 – 120 | 76 | 30 – 120 | 146 | 30 – 120 | 1299 | 28 – 253 |
| Arginine | 4 | 2 – 15 | <1 | 2 – 20 | 3 | 2 – 10 | 3 | 0 – 19 |
| Ornithine | 14 | 5 – 15 | 5 | 2 – 10 | 3 | 2 – 10 | 10 | 0 – 30 |
| Lysine | 172 | 5 – 20 | 27 | 5 – 30 | 76 | 5 – 30 | 221 | 0 – 182 |
| Cystine | 24 | 5 – 35 | 12 | 5 – 25 | 13 | 5 – 20 | 17 | 0 – 36 |
| Methionine | * | * | 11 | 3 – 15 | 9 | 3 – 10 | 19 | 2 – 20 |
| Taurine | 607 | 30 – 55 | 147 | 30 – 105 | 429 | 40 – 200 | * | * |
| Phenylalanine | 17 | 5 – 20 | 17 | 5 – 15 | 15 | 4 – 15 | 40 | 5 – 37 |
| Tyrosine | 51 | 5 – 30 | 25 | 5 – 15 | 25 | 5 – 20 | 128 | 10 – 72 |
| Tryptophan | 16 | 1 – 15 | <1 | 1 – 5 | 10 | 1 – 6 | * | * |
| Histidine | 314 | 50 – 155 | 203 | 100 – 300 | 139 | 100 – 300 | * | * |
| Aspartate | 105 | 10 – 45 | 50 | 10 - 35 | 39 | 10 – 30 | * | * |
| Glutamate | 14 | 2 – 15 | 24 | 0 - 3 | 10 | 0 - 3 | 59 | 0 – 69 |

Amino acid concentrations elevated above the reference range are shown in orange and those below the reference range are shown in blue. Appropriate reference range employed at the time of analysis are shown adjacent to each result. * Not quantified at this time.
