# Supplementary material for: Mutations in SLC25A22: hyperprolinaemia, vacuolated fibroblasts and presentation with developmental delay
Source: J Inherit Metab Dis. 2017 Mar 2;40(3):385–94. doi: 10.1007/s10545-017-0025-7 (PMC5393281; doi:10.1007/s10545-017-0025-7)
Supplement: Supplementary file 7 — (DOCX 13 kb) [file 10545_2017_25_MOESM7_ESM.docx]

**Supplementary Table 5:** **CSF amino acids measured in patients 1 and 5.**

|  | Patient 1 (µmol/L) | Patient 5 (µmol/L) | Reference range (µmol/L) |
| --- | --- | --- | --- |
|  | **2 months 19 days** | **4 years 6 months 25 days** |  |
| Glutamate | 1 | 1 | 5 – 17 |
| Threonine | 56 | 36 | 10 – 45 |
| Serine | 72 | 31 | 10 – 81 |
| Glycine | 4 | 4 | 4 – 14 |
| Proline | 12 | <1 | 0 – 1 |
| Leucine | 22 | 14 | 4 – 18 |
| Isoleucine | 11 | 6 | 3 – 8 |
| Valine | 26 | 16 | 5 – 25 |
| Alanine | 31 | 25 | 16 – 36 |
| Glutamine | 621 | 458 | 420 – 600 |
| Arginine | 22 | 19 | 15 – 40 |
| Ornithine | 7 | 3 | 3 – 13 |
| Lysine | 27 | 18 | 10 – 32 |
| Methionine | 3 | 2 | 2 – 6 |
| Taurine | 9 | 5 | 3 – 10 |
| Histidine | 14 | 14 | 3 – 18 |
| Tryptophan | 2 | 3 | 1 – 3 |
| Phenylalanine | 9 | 7 | 5 – 15 |
| Tyrosine | 21 | 10 | 5 - 15 |

Amino acid concentrations elevated above the reference range are shown in orange and those below the reference range are shown in blue.
